# Imaging methods used in the assessment of environmental disease networks: a brief review for clinicians

**DOI:** 10.1186/s13244-019-0814-7

**Published:** 2020-02-07

**Authors:** Aime Cedillo-Pozos, Sergey K. Ternovoy, Ernesto Roldan-Valadez

**Affiliations:** 1 Directorate of Research, Hospital General de Mexico “Dr. Eduardo Liceaga”, Mexico City, Mexico; 2 Department of Radiology, I.M. Sechenov First Moscow State Medical University (Sechenov University), Moscow, Russia; 3 A.L. Myasnikov Research Institute of Clinical Cardiology of National Medical Research Center of Cardiology of the Ministry of Health of Russia, Moscow, Russia

**Keywords:** Air pollutants, Diagnostic imaging, Environmental health, Environmental illness

## Abstract

**Background:**

Across the globe, diseases secondary to environmental exposures have been described, and it was also found that existing diseases have been modified by exposure to environmental chemicals or an environmental factor that has been found in their pathogenesis. The Institute of Medicine has shared a permanent concern related to the nations environmental health capacity since 1988.

**Main body:**

Contemporary imaging methods in the last 15 years started reporting alterations in different human systems such as the central nervous system, cardiovascular system and pulmonary system among others; evidence suggests the existence of a human environmental disease network. The primary anatomic regions, affected by environmental diseases, recently assessed with imaging methods include Brain (lead exposure, cerebral stroke, pesticide neurotoxicity), uses MRI, DTI, carotid ultrasonography and MRS; Lungs (smoke inhalation, organophosphates poisoning) are mainly assessed with radiography; Gastrointestinal system (chronic inflammatory bowel disease), recent studies have reported the use of aortic ultrasound; Heart (myocardial infarction), its link to environmental diseased has been proved with carotid ultrasound; and Arteries (artery hypertension), the impairment of aortic mechanical properties has been revealed with the use of aortic and brachial ultrasound.

**Conclusions:**

Environmental epidemiology has revealed that several organs and systems in the human body are targets of air pollutants. Current imaging methods that can assess the deleterious effects of pollutants includes a whole spectrum: radiography, US, CT and MRI. Future studies will help to reveal additional links among environmental disease networks.

## Key points

- Existing diseases have been modified by exposure to environmental chemicals.
- Air pollutants are derived from anthropogenic and natural sources.
- Particulate matter can pass from alveoli to blood circulation to body systems.
- Medical imaging has revealed deleterious effects in nervous, cardiovascular and pulmonary systems.
- Imaging methods may allow understanding of harmful mechanisms of environmental chemicals.

## Introduction

In recent years, across the globe, new diseases secondary to environmental exposures have been described, and it was also found that existing diseases have been modified by exposure to environmental chemicals or an environmental factor that has been found in their pathogenesis [1].

**Fig. 1:**
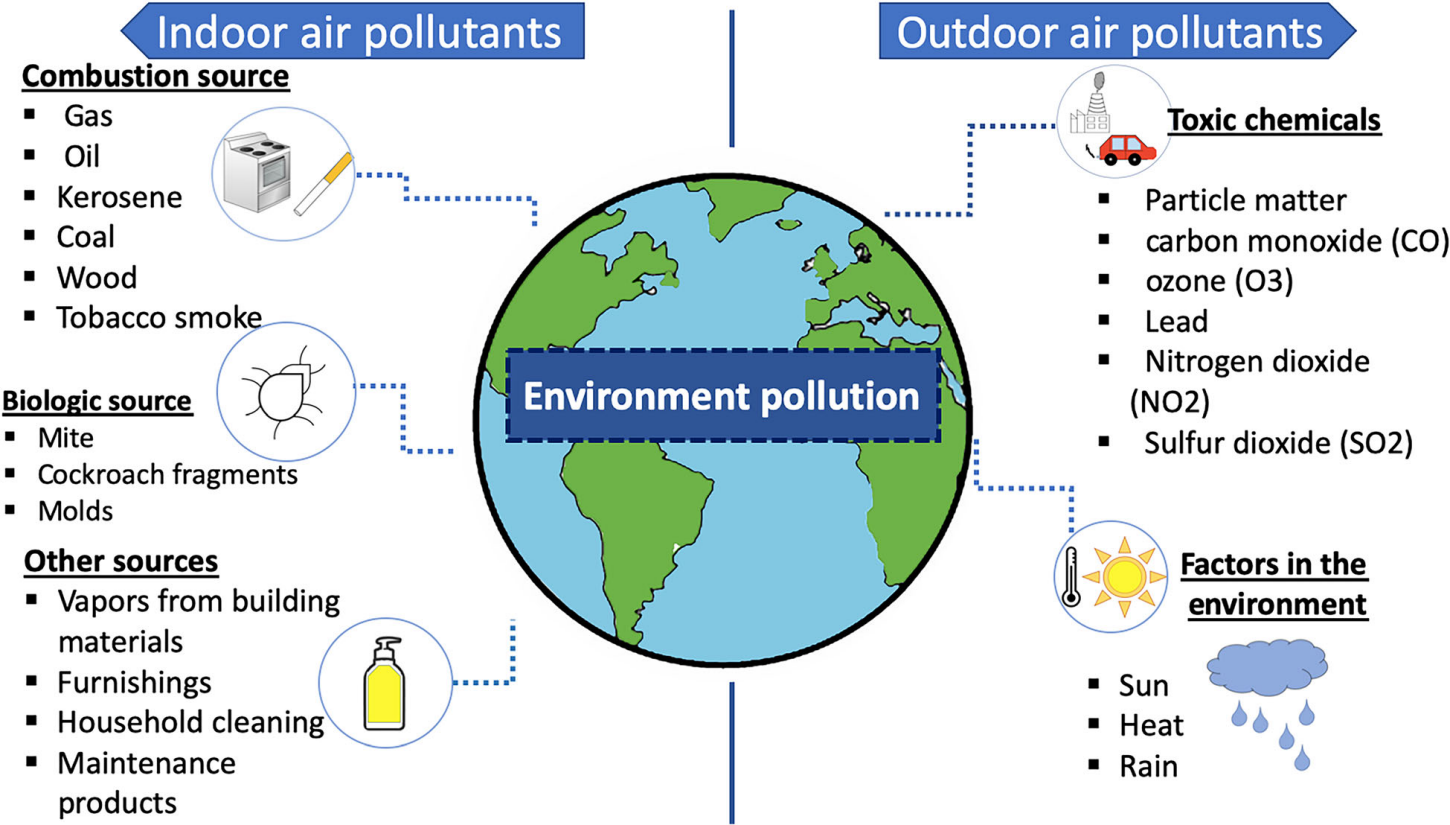
Relationships between air pollutants and environmental diseases

Environmental diseases have been differentiated from pollution-related diseases. The first one arises as a result of direct exposure of the patient to environmental pollutants; this includes diseases caused by exposure to toxic chemicals, substance abuse as well as exposure to physical environmental factors such as UV radiation and familiar history, and the second one: pollution-related diseases are based on the exposition to water, air and soil toxins. Both of them are considered environmental diseases [2]. Figure 1 shows a diagram that explains air pollutants and environmental diseases.

A permanent concern related to the nations environmental health capacity has been shared by the Institute of Medicine since 1988, when it expressed that “the removal of environmental health authority from public health agencies has led to fragmented responsibility, lack of coordination and inadequate attention to the public health dimensions of environmental issues” [3].

Contemporary imaging methods in the last 15 years started reporting alterations in different human systems such as the central nervous system [4], cardiovascular system [5] and pulmonary system [6] among others; evidence suggests the existence of human being environmental disease network [1].

There are no global or local strategies for the identification of the danger of environmental pollutants, the measurement of the exposure of the population or the monitoring of health conditions related to the pollutant. For all the reasons mentioned above, we aimed to present a brief review of the current imaging methods used in the regional assessment of human environmental diseases with the organs or systems that are involved.

### Chemical substances linked to environmental diseases

Air pollution is a combination of diverse gases and particles; pollutants that represent a significant public health problem involve particulate matter (PM), a mixture of elemental and organic carbon, polycyclic aromatic (PAH) and metals hydrocarbons, ozone (O_3_), carbon monoxide (CO), lead, sulfur dioxide (SO_2_) and nitrogen dioxide (NO_2_) [7].. They are derived from the various natural and anthropogenic origin. Pollutants are divided into indoor air pollution and outdoor air pollution. Although out-of-doors contamination can get into the indoor environment, there are also exclusive pollutants of indoor environment as a result of combustion such as gas, coal, kerosene, oil, wood and tobacco and the biologic contamination like cockroach fragments, moulds, vapours from furnish and building materials and cleaning products [8].

Another air pollutant is environmental tobacco smoke which could produce a pro-atherosclerotic state meaning the thickening of intima and media in the aorta and carotid arteries, as well as the worsening of pre-existing atherosclerotic arteries [9].

### Human organs and systems involved in environmental diseases

Environmental pollution has been studied for its potential health damage as particulate matter get into the alveoli of the lung then come into the blood circulation due to its size and chemical activity [10]; all these particles travel all way to the brain and cause severe effects on the central nervous system (CNS). Alterations on CNS modulate some responses of the cardiovascular, pulmonary and immune systems facing toxic chemicals exposure [8]. Contamination has been associated with pro-thrombotic and coagulant changes, endothelial dysfunction, vasoconstriction, increased blood pressure, systemic inflammatory and oxidative stress responses, autonomic imbalance and arrhythmias [11].

There are specific conditions associated with prenatal or postnatal exposure to pollutants like a decreased IQ in children, autism spectrum disorders, neurodegenerative diseases such as Parkinson disease and Alzheimer disease. All of them due to changes during neurodevelopment in children and neurologic lesions in adults such as a continuous state of brain inflammation, microglial activation and white matter changes; other diseases linked to air pollution are multiple sclerosis and stroke [8, 12] (Table 1).

**Table 1:** Anatomic regions affected by environmental diseases that have been assessed with imaging methods

Region	Disease	Imaging method	Findings
Brain	Lead exposure	MRI	Decreased NAA/Cr ratios in grey matter wich suggest neuronal loss. Finding were more remarkable in frontal regions [13].
Brain		DTI	DTI shows abnormalities in radial diffusivity which means changes in myelin sheath thickness and organisational characteristics.
Brain	Cerebral stroke	Carotid ultrasonography	Increased carotid intima-media thickness, decreased carotid arteries distensibility, early atherosclerotic lesions [9].
Brain	Pesticide neurotoxicity	MRS	Regional elongation in the cerebral surface with changes in the gyrus rectus, superior frontal gyrus, cuneus and precuneus along the mesial wall of the right hemisphere. Anatomical alterations in the mesial and dorsal surfaces of the left superior frontal gyrus [4].
Thorax–lungs	Smoke inhalation	Radiography	Chest radiography shows three stages: 1. The acute stage < 24 h after exposition: normal characteristics of the lungs. 2. The subacute stage 2–5 days after exposition: manifests as pulmonary oedema, atelectasis, pulmonary micro-embolism, and adult respiratory distress syndrome (ARDS). 3. The delayed stage > 5 days after exposition: pneumonia and pulmonary thromboembolism [6].
Lungs	Organophosphates poisoning	Radiography	Lung oedema that could appear within 24 hours after exposition [14].
GI	Chronic inflammatory bowel disease	Aortic ultrasound	Increased aortic intima-media thickness [9].
Heart	Myocardial infarction	Carotid ultrasound	Increased carotid intima-media thickness [9].
Arteries	Artery hypertension	Aortic and brachial ultrasound	Higher stiffness and lower elasticity [9].

In several countries around the world, mostly those with a high level of urbanisation and industrialisation present another health problem derived from noise. It could be defined as the propagation of sound capable of cause damage in humans [15]. There are different sources of noise, such as music, traffic, air transportation, construction and industrial activities [15]. The health effects of noise are divided into auditory effects like hearing loss which has side effects such as lower working concentration and non-auditory effects which include anxiety syndrome, sleep dysfunction, cognitive impairment and there is report of cardiovascular disease like arterial hypertension and myocardial infarction [16, 17]. Unfortunately, there are no imaging methods used yet for screening or diagnosis of acute or chronic exposure to noise.

### Cardiovascular conditions linked to environmental pollution exposure

Although the mechanisms responsible for the cardiovascular effects are not well elucidated, studies through the last decades have shown that particulate matter with diameter < 10 μm are linked with higher mortality and morbidity from cardiovascular diseases [18, 19]. In 2001, a study by Peters A. et al. suggested that the exposition to elevated concentrations of fine particles increases the probability of myocardial infarction in a few hours [20].. Deaths due to air pollution episodes have been well documented through human history; Belgium in 1930, 63 people died during a 5-day fog, most of them with previous known heart and lung diseases; Pennsylvania in 1948, 20 people died and 7000 were affected with acute illness; London in 1952, 4000 people over 45 years old died [21]. Nowadays, there is evidence that living even at much lower particle concentrations may contribute to long-term risks of death from heart disease [22]; all these are important because cardiovascular disease is considered the most common cause of mortality in developing and developing countries, where the mortality is 50% [23].

#### Ischemic heart disease

The ACS study demonstrated that chronic exposure to elevated PM2.5 levels elevates the risk of ischemic heart disease [24]. Other conditions associated with exposure to contamination are acute myocardial infarction, which has been reported by different authors [20, 25, 26], as well as a higher probability of presenting a subsequent myocardial infarction, hospital admission for congestive heart failure and, in the worst-case scenario, death [25]. A study in which coronary angiography was performed to patients who previously presented a myocardial infarction episode found a relation between PM air pollution and ischemic cardiac events in those patients whom already have obstructive coronary atherosclerosis in at least one vessel [24]. Patients with a preexisting coronary artery disease present a higher risk of an acute ischemic event within days or even hours [27].

#### Heart failure

The exposure to environmental pollution is linked with the increase of the calcium in the coronaries [28], cardiac remodelling in addition to right and left ventricular hypertrophy [29]; these changes increased the heart failure admissions [30].

#### Cerebrovascular disease

Two studies in Seoul Korea found out that elevated air pollution concentrations enhance the stroke mortality; the association was with ischemic stroke but not with the hemorrhagic [31]. In Helsinki, Finland, higher mortality was found during warm seasons, which demonstrates that climate has a role to play on environmental diseases [32].

#### Peripheral arterial and venous diseases

There is not enough information about the links between pollution exposure and peripheral vascular diseases; however, a study in Italy found 70% risk of deep vein thrombosis in long-term elevation of PM_10_ levels [33]; the increases of coagulation and prothrombotic state could be the mechanism responsible for damage of the venous system and arterial cardiovascular system [11].

## Environmental lung diseases: imaging findings

Although many diseases related to the environment have been described in the last years, environmental lung diseases still predominate, and different authors are continually describing them. In recent years, radiography and computed tomography have been used as diagnostic methods of choice for different pneumoconioses and exposure to toxic chemicals, such as PM2.5, lead and tobacco smoke [6, 34]. We describe the CT and chest radiography findings in different pneumoconiosis.

### Silicosis

A fibrotic condition of the lungs that occurs after many years of exposition to crystalline silica. It is rare to find by chest radiography, but at the initial stages of silicosis, high-resolution computed tomography scans show linear and nodular opacities distributed in both lungs [6], which in addition to clinical presentation, is an essential tool for the diagnosis of these pneumoconioses.

### Coal pneumoconiosis

This lung disease is caused by the exposition to a dust mixture of mainly coal, but also kaolin, mica and silica. Dust lung disease caused by coal includes chronic bronchitis, emphysema and diffuse fibrosis. The radiological findings are interstitial opacities in the superior zones of the lung < 1 cm; it is also possible to use CT scans where small areas of low attenuation could be observed [35, 36].

### Graphite pneumoconiosis

Graphite exposure and coal miners present similar radiographic and pathologic changes. CT findings are septal thickening with significant opacities and small nodular hyperattenuating areas [37].

### Asbestosis

Pulmonary asbestosis is diffuse interstitial fibrosis as a result of the exposition dust containing asbestos; the radiographic characteristics are small, irregular or regular opacities in the inferior zones of the lungs, pleural thickening or pleural plaques. CT manifestations are honeycombing, parenchymal bands and subpleural curvilinear lines; in the interstitial space, it presents as thickened intralobular core or irregularly thickened septae [37–39].

### Talcosis

Talcosis presents three types of lesions: ill-defined nodular lesions, diffuse interstitial pulmonary fibrosis and foreign body granulomas; chest radiography could be used as a diagnostic method with the following findings: large opacities, nodular or diffuse interstitial or a mixture of linear and nodular patterns [37]. Thin-section CT has high sensitivity for this kind of pneumoconiosis, which shows subpleural and small centrilobular nodules, and conglomerate masses with heterogeneous high-attenuation foci [40].

### Welder’s lung/siderosis

Lung macrophages characterise it with iron oxide accumulation. Findings on plain films include perihilar small nodules more notorious in the middle third of the lungs [41]. CT characteristics comprise blurred micronodules with diffuse distribution sometimes appearing as fine branching lines. The lung lesions go from micronodules showing the centrilobular distribution in a less affected lung to a network pattern formed by micronodules or an area of ground-glass attenuation [37].

### Berylliosis

Beryllium disease has two presentation forms: the first one an acute pneumonitis, after a brief exposure to a high level of beryllium, and the second one, a chronic granulomatous pulmonary disease for considerable accidental exposure. CT presents higher sensitivity than chest radiography to diagnose beryllium disease, showing well-defined parenchymal nodules and septal lines, often disseminated along with the interlobular septa or bronchovascular bundles [37].

### Hard metal lung disease

Hard metal lung disease (HMLD) clinical manifestations include asthma, upper respiratory tract irritation and an interstitial lung disease manifested by chronic cough or dyspnea. Findings in chest radiography, especially in advanced disease include diffuse nodular, small reticular patterns and cystic spaces. CT findings consist of consolidation on a panlobular or multilobular scale, bilateral opacities similar to ground-glass and traction bronchiectasis or extensive reticular opacities [41].

## Imaging methods used in the assessment of environmental diseases

Latest advances in imaging methods have opened unrivalled access to the study of the chemical, environmental mechanisms that cause alterations in different body systems (Fig. 3).

Imaging methods allow a more precise and accelerated diagnosis of numerous diseases related to the exposition of air pollutants; investigators from different parts of the world are using imaging methods such as plain radiography [42], ultrasonography (US), echocardiography [9], computed tomography (CT) [43] and magnetic resonance imaging (MRI) [7], to help us understand environmental disease pathophysiology and evolution (Table 2).

**Table 2:** Imaging methods used in the evaluation of diseases associated with exposure to environmental pollution

Imaging method		Environmental disease
Radiography		1. Smoke inhalation
USG	Carotid arteries ultrasonography	2. Myocardial infarction
	Brachial artery ultrasonography	3. Arterial hypertension
	Aorta ultrasonography	4. Chronic inflammatory bowel disease
Echocardiography		5. Heart failure
Computed tomography		6. Hypersensitivity pneumonitis
Magnetic resonance imaging	Anatomical Magnetic resonance (AMR)	7. Changes in neurodevelopment caused by pesticide exposure
	Diffusion tensor imaging (DTI)	8. Changes in neurodevelopment caused by lead exposure
	Functional magnetic resonance (fMRI)	9. Tobacco smoke neurotoxicity in children
	Magnetic resonance spectroscopy	10. Pesticide neurotoxicity

### Radiography

Despite the technological advances in the imaging methods, chest radiography continues being the imaging method of choice for lung diseases, and environmental diseases are not de exception, although the other imaging techniques also will have a significant role in the future for lung diseases [42].

The International Labour Organisation (ILO), proposed in 1930 the International Classification of Radiographs and now has been adopted universally to provide the meaning of radiographic abnormalities in the chest caused by inhaling dust [42]. The exposition to different environmental conditions, such as the inhalation of toxic agents or atmospheric pressure changes, which could cause pneumoconioses such as classic Hard-metal pneumoconiosis, asbestosis, berylliosis, coal worker and silicosis [41].

### Ultrasonography

In a review by Paweł Gać et al., they identify the ultrasound as the primary method to diagnose cardiovascular system pathologies [9]. Different authors have associated cardiovascular changes related to tobacco smoke exposure using carotid ultrasound. They found increased carotid intima-media thickness and decreased carotid arteries distensibility [44], which could explain why these patients presented increased cardiovascular risk [9].

### Computed tomography

**Fig. 2:**
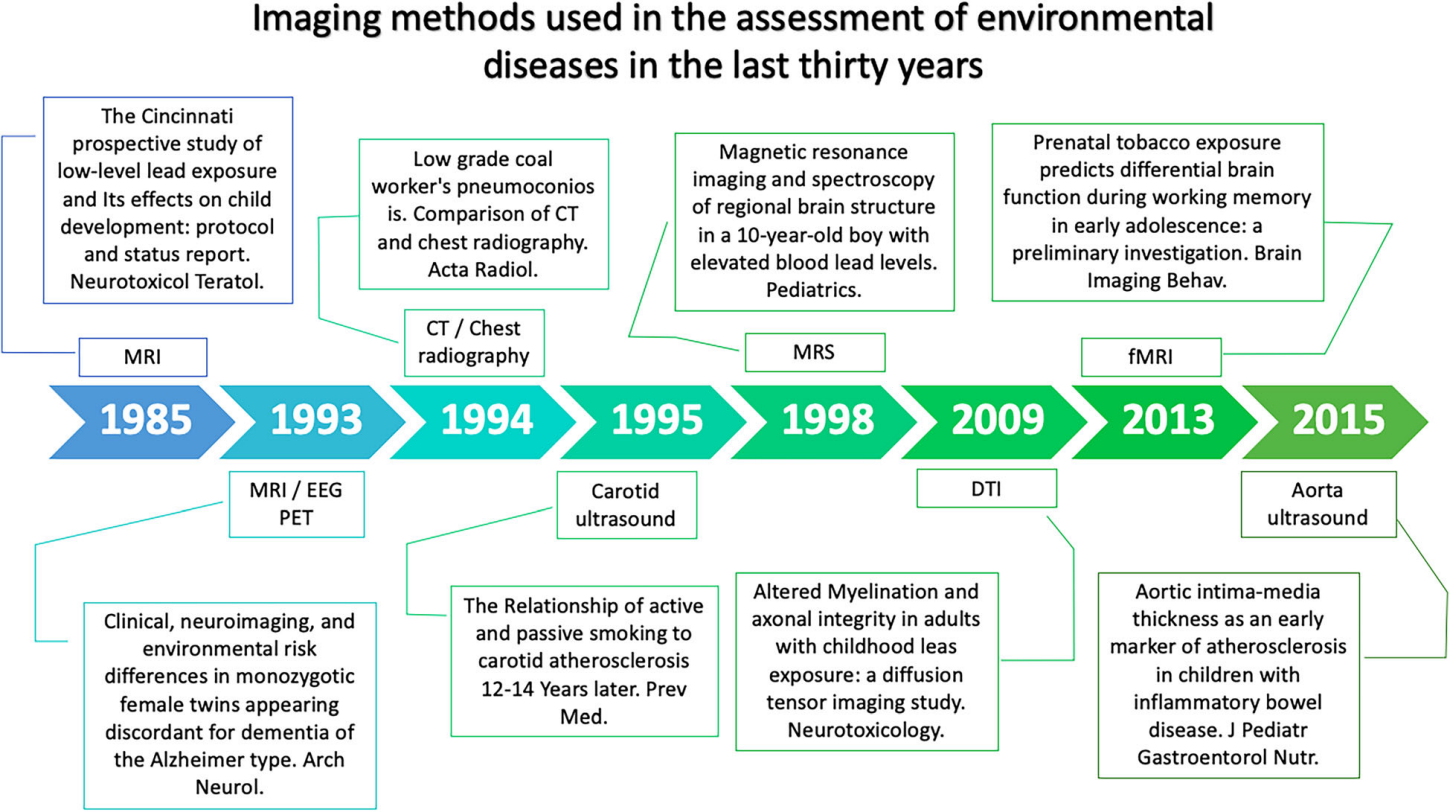
Timeline representing the evolution of imaging methods in the study of environmental diseases

CT essential in the imaging assessment of environmental and occupational lung diseases. High-resolution computed tomography (HRCT) shows higher sensitivity than plain film in the diagnosis of lung abnormalities in silicosis, asbestosis and other pneumoconioses [34]. Because lungs represent the more affected organs, multiple studies have reported CT changes in the lungs from multiple diseases. Such as silicosis in which CT scans show the progression to a confluence of nodules with or without progressive massive fibrosis [37, 41] (Fig. 2).

### Magnetic resonance imaging

MRI permits the investigation of brain structure and function in-vivo, using the body’s natural magnetic properties from hydrogen atoms of water to analyse images of the body [4]. It has been used to relate exposure to environmental chemicals with neurodevelopmental disorders (for example, language impairment, speech, learning disabilities and attention deficit, autism spectrum disorders and hyperactivity disorder) in childhood, and neurodegenerative diseases in elderly (PD or AD), among others [8].

### Anatomical MRI

Anatomical MRI discriminates between grey matter, white matter and cerebrospinal fluid and creates a static limit of the morphological brain, and it is used to assess the volume of specific regions of the brain [4, 45, 46]. MRI findings are relevant biomarkers in many neurologic conditions, and neuropsychological processes such as memory [47, 48], which may reveal hints to understand how environmental toxicants elicit neurodevelopmental disorders [49]. A pilot study in 2012 performed brain MRI in children with the antecedent of elevated prenatal exposure to organophosphates pesticide and demonstrated regional enlargements of the posterior middle temporal, superior temporal and inferior postcentral gyri bilaterally, also in the superior frontal cuneus, gyrus, precuneus and gyrus rectus. The children who presented these findings were associated with a lower IQ [50], similar to previous associations of cognitive impairment in children and prenatal exposure to CPF [51].

### Diffusion tensor imaging

Diffusion tensor imaging (DTI) is one of the more contemporary methods to visualise brain structure [52, 53]. However, DTI provides data to evaluate not only white matter integrity but also fibre connectivity [49]. For example, studies have used DTI values to derive almost a dozen of quantitative biomarkers and have also tracked the changes in signal intensity to understand the progression of the glioblastoma [54, 55].

### Functional MRI

Functional MRI employs changes in oxygenation states of blood to grant an indirect assessment of neuronal activity [56]. Functional magnetic resonance imaging (fMRI) used on children who were prenatally under the exposition of tobacco smoke to establish the impact of it, which is known is linked with changes in neurocircuitry that support attention and learning skills [57]. There have been findings of more activation during a memory test in the inferior parietal regions contrasting with unexposed children who presented more activation in bilateral inferior frontal regions. This activation suggests that children use different brain regions while working on a memory test depending if they were exposed or unexposed; this as a compensatory process to neurotoxic exposures [58]; these findings may explain why learning, emotional and behavioural problems may occur more frequently in children with poor working memory [59].

### Magnetic resonance spectroscopy

**Fig. 3:**
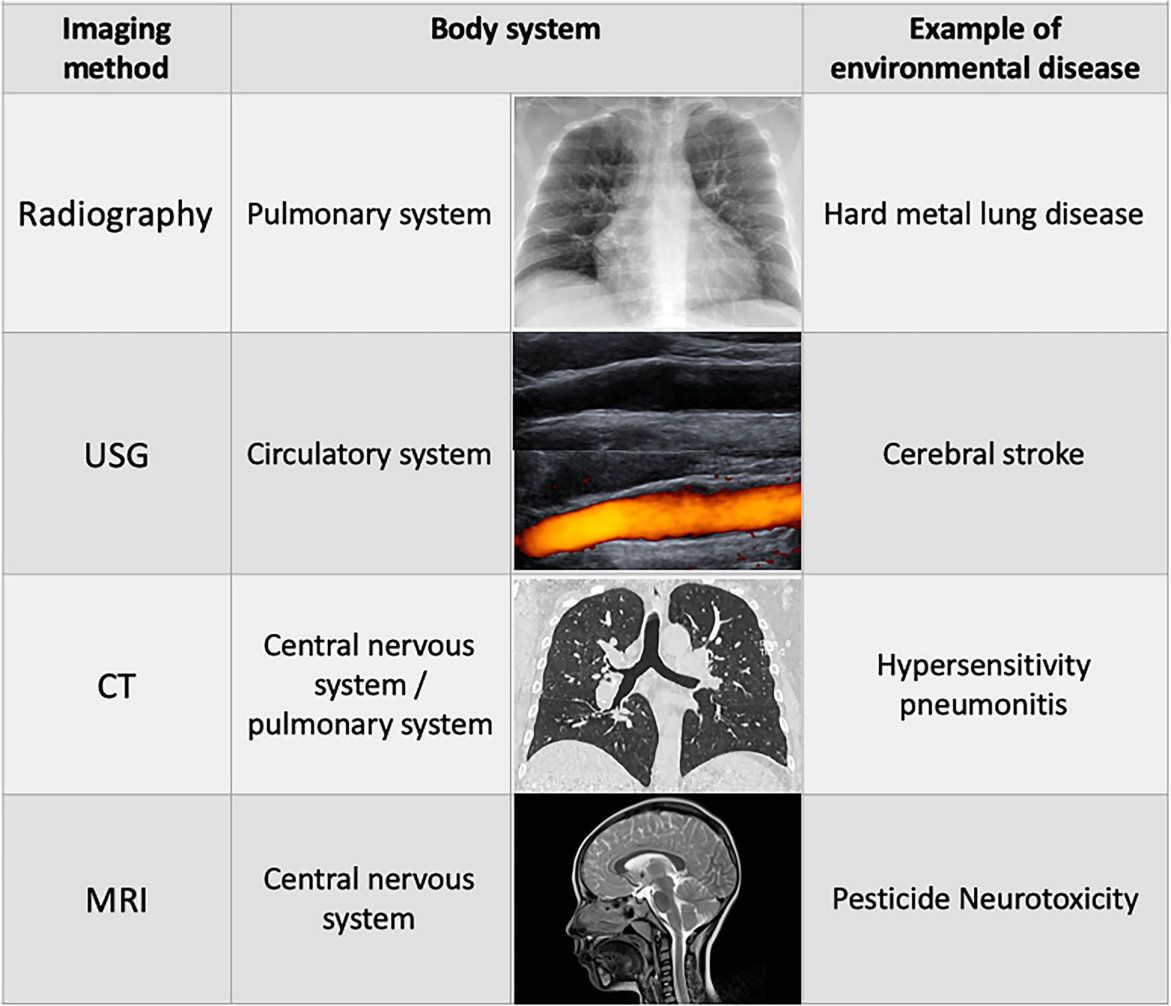
Imaging methods that have been used in the assessment of environmental diseases in the last 30 years

Magnetic resonance spectroscopy (MRS) grant in vivo analysis of cerebral metabolites that establish chemical content in some brain regions, some of the metabolites more frequently studied is choline, *N*-acetyl aspartate (NAA), creatine and glutamate (Glu) [60]. Although the most common use of MRS has been in the evaluation of glioblastoma’s survival [61], and epilepsy [62], recent applications of the MRS for environmental diseases have identified neurodevelopment variations due to prenatal exposure [4]. Studies about prenatal exposition to environmental pollution and the exposition during the first year of life are essential because of the importance of brain development during this time [63]. MRS has been used to study neurodevelopmental syndromes like autism spectrum disorder; it is characterised by glutamate and glutamine alterations in the cortex and the basal ganglia in adults and children [64] (Fig. 3).

## Environmental factors and the development of cancer

The development of cancer is the result of an interaction of multiple risk factors; the individual factors such as genetic predisposition and modifiable risk factors which include the consumption of alcohol, tobacco smoke and infections; and the environmental risk factors like the exposure to ultraviolet light, ionising radiation, gaseous pollutants, particulate pollutants, noise and heat [65, 66]. According to statistics by the WHO, 35% of deaths due to cancer had a history of modifiable risk factors that could be detected by screening, with the use of imaging methods or other screening methods [67, 68]. It is necessary to establish control strategies because even though it is well known that environmental pollution is causing several health problems, many countries do not have control strategies or they have them, but these strategies are not implemented aggressively. Unfortunately, people with hard exposition to all these pollutants are those with the lowest incomes; this related to less education and lower economic resources in addition to more polluting industries located in poorer neighbourhoods [69]. As a result of all these factors, cancer diagnosis is growing all around the world (Table 3 shows the use of imaging methods in the screening of patients with cancer risk for exposure to environmental factors).

**Table 3:** Use of imaging methods in the screening of patients with cancer risk for exposure to environmental factors

Environmental risk factors	Type of cancer	Imaging methods used for screening
Exposure to magnetic fields	Leukaemia	Mammography as screening for breast cancer
	Breast cancer	
Ionising radiation ▪ X-rays ▪ Radiotherapy ▪ Nuclear industry	Leukaemia	Ultrasound as an initial method of screening for thyroid and liver while X-ray is the best option for osteogenic sarcoma
	Thyroid cancer	
	Liver cancer	
	Osteogenic sarcoma	
Ultraviolet radiation	Melanoma and squamous cancer	N/A
Tobacco smoking	Lung cancer	Chest film and CT are the screening methods of choice for those organs affected by tobacco smoke, another tool used in these cases is the bronchoscopy
	Larynx cancer	
	Throat cancer	
	Bladder cancer	
Ambient particulate matter pollution	Tracheal cancer	
	Bronchus cancer	
	Lung cancer	
	Mesothelioma	
Occupational exposure	Lung cancer	CT is used as the initial method
	Lymphomas	
	Brain tumours	
Infections (*H*. *pylori*)	Gastric cancer	Endoscopy and colonoscopy with gastric tissue biopsy

## The role of CAD and AI in the assessment of environmental diseases

The use of computed techniques with artificial intelligence may help the prevention of many diseases by creating networks of cause and effect, which will help us predict better when a patient is at risk of suffering from a disease these tools are named computer-aided design (CAD). Artificial intelligence (AI) is nowadays a promising tool in medical practice. AI applications in health have included predictive models to identify populations at risk [70]; some of them presented as mobile health care delivery and medical imaging interpretation apps [70–72]. The recognition of all the environmental factors and causes of exacerbation in lung diseases using an artificial intelligence application was described in a publication by Philip Haber et al. [73]. The system used in that study mixed both inference process and knowledge, including different factors such as the specifics of the diagnosis, time course of the illness, time of environmental factor exposure, measures of likelihood exposures and other effects. There is evidence that it is possible to use an AI expert system to identify possible risk factors in a period of work to detect adverse health effect [73].

## Radiology contribution to environmental illness

The use and development of imaging techniques for diagnostics and therapies have opened a new perspective on medicine, but it also has some side effects on human health, some of them for the exposition to radiation and some others because of the use of enhanced techniques.

A diverse dosage of ionising radiation (IR) is used in medical imaging and treatment, from very low dose as X-ray imaging which uses a range of 0.005 to 1.5 mGy depending on the imaged body part to significant doses in Computed tomography scans used in the range of 2 to 65 mGy per procedure [74]. Another use of ionising radiation in medical terms are the treatments for non-malignant disorders such as degenerative or inflammatory diseases in which the dose is usually higher than 3–30 Gy, and for cancer therapy the dose varies between 16 and 75 Gy, depending on size, type of tumour [74]. Nevertheless, IR procedures are associated with tissue damage with severe consequences; most of them in paediatric patients (< 3 years old). These adverse effects could be intellectual and memory impairment, visual and hearing loss; these children also have shown a progressing mental impairment just as Alzheimer’s disease [75]. All these changes suggest an impact on neurogenesis which is still ongoing in paediatric patients [76]. Another side effect of radiation reported is the brain injury that may result in calcifications visible as an increased of magnetic resonance signal intensity [77].

The development of the medical imaging techniques has improved the diagnostic modalities in the area of medicine, and one of the most important in the recent decades is MRI; it has proved to be safe and effective, even though recent studies have shown long term consequences when MRI is used with enhanced components such as gadolinium [78]. It has been observed in patients with previous gadolinium administration a high signal intensity in the dentate nucleus and globus pallidus in the brain, with no association with renal function [78]. However, the association between gadolinium use for diagnostic techniques and these findings remains unclear [79]. Another condition associated with the use of gadolinium was described for the first time in 1997 as a nephrogenic fibrosing dermopathy; later in 2000, on a report about a scleromyxoedema like disease in patients with renal dialysis [80]. Similar reports were published in the last two decades, this new disease associated with gadolinium was finally named nephrogenic systemic fibrosis (NSF), in which there are fibrotic changes in several organs such as the muscles, heart, liver and lungs; in more than 50% of the patients, it is progressive and severe [81, 82]. It has been reported in adults and children with no age predilection [83]. The prevalence of NSF was calculated in patients with renal failure to be 3–7% [84] (Fig. 3).

## Specific reporting guidelines for diseases due to environmental exposure

**Fig. 4:**
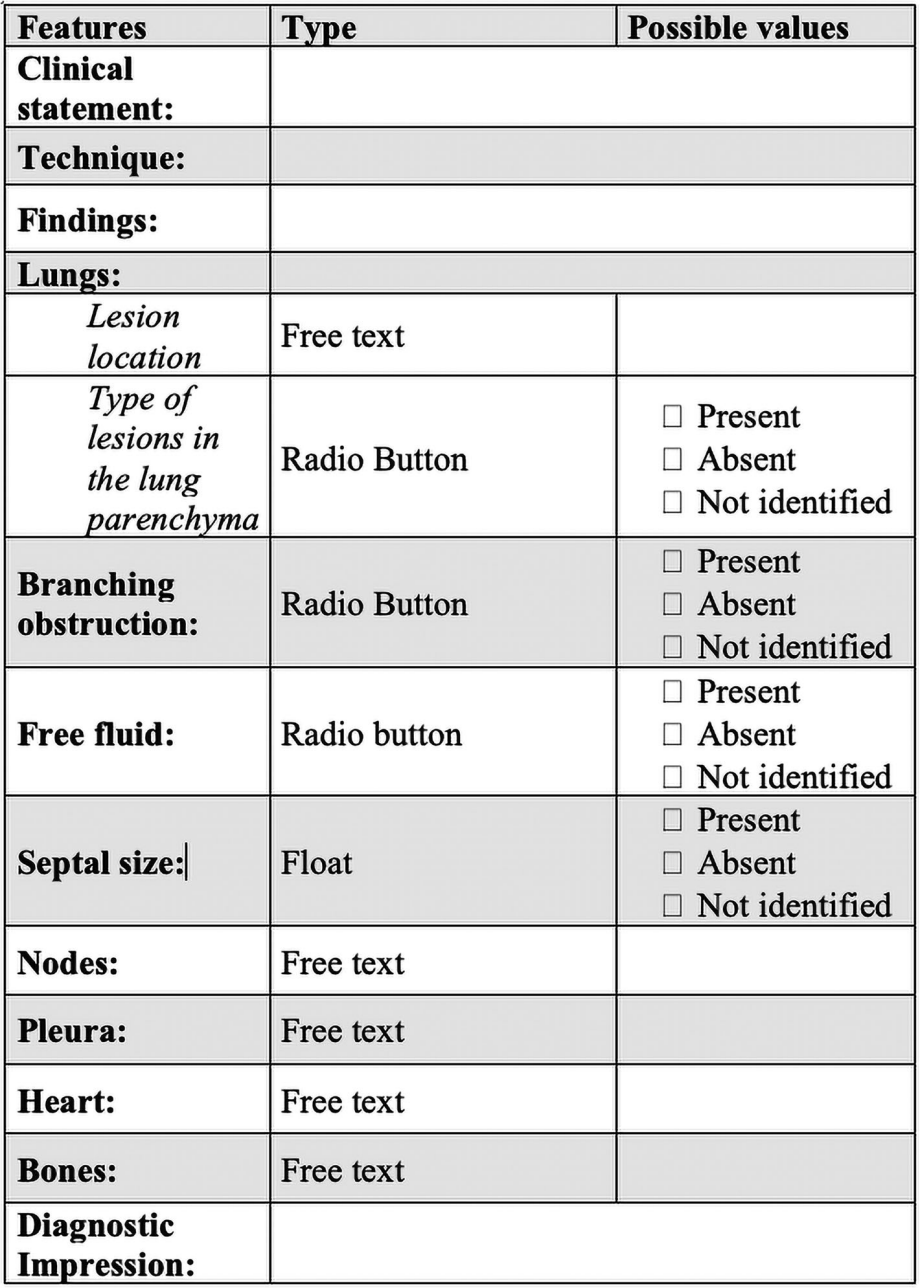
Example of a reporting guideline for CT thorax examination in the assessment of environmental disease

Since 2011, The European Society of Radiology and Radiological Society of North America through their journals encouraged authors to provide guidelines to standardise the form and language on the structured reports to specify how to describe the radiologic findings of a specific region disease [85, 86]. In the elaboration of reporting guidelines, it is essential to understand two concepts: structured reports and common data elements (CDE); both terms are different but overlaying: while structure reports describe information like technique, clinical information, correlation, findings, diagnostic impression and additional information necessary to make clear the description of the disease, CDE is a mixture between specific observations and its possible values; together, they form the ideal radiologic report of a specific disease [87]. A possible example could be done with occupational lung diseases for which CT is used as a method of choice for its evaluation. The structure report of an occupational disease should describe all the imaging characteristics such as linear or nodular opacities, localisation of the lesion, size of the opacities and if there is or not septal thickening; all these features finally constitutes the CDE. It also should include the description of the adjacent organs, for example, pleura, nodes and heart [88] (Fig. 4 depicts an example of a hypothetic reporting guideline for CT thorax examination in the assessment of environmental disease).

## Future directions

Neuroimaging techniques have been studying brain development under exposition to environmental pollution in the last three decades. There are two main aspects of neuroimaging research in this field: *prenatal exposure to drugs* and *mood disorders in pregnant women*; in the first one, the use of active medication should be better controlled in future studies because it represents a confounding factor [89–92].

The second aspect has the limitation that multiple drug usage acts as a significant limitation for the characterisation of brain changes associated with a specific drug. To tease drug-specific effects is necessary to run multivariate modelling of all implicated drugs with larger sample sizes to have enough statistical power [93–96].

Readers should remember that brain alterations linked to environmental pollutants are nowadays detected in prenatal and postnatal studies; the alterations may change depending on the age of the patient and vary between boys and girls. The primary evidence that imaging studies can reveal is the association between behavioural outcomes with early brain alterations [97]. Two important questions for the future are:

“*What do the observed early brain alterations tell us about future behaviour*”? The answer to this question requires longitudinal imaging studies of behavioural outcomes started during infancy. Also, “*What can we do to modify the aberrant brain growth, thus improving behavioural outcomes*”? Evaluating the effects of intervention trials using images based on quantitative biomarkers might help to answer this question [97].

Prospective studies combining epidemiological and neuroimaging variables may (1) use repeated brain MRI to understand the connection of traffic-related air pollution in brain development changes and ageing diseases, (2) to evaluate children at risk with early MRI and (3) to discover new complex associations between fMRI data, acute exposure to TRAP and neuropsychological confounders such as cognition, psychomotor and social-emotional changes, in the induction of brain changes [7, 98].

For the other organs and systems, the next step in the study of the environmental pollutants and its relationship with human health will be to increase studies size to understand better and detect smaller associations between health and pollution exposure [63]. It is also vital to determine susceptible individuals or vulnerable populations; to make this possible, further studies should add computational methods to enhance our current knowledge [63], as well as imaging methods, get better in the assessment of environmental diseases.

## Conclusions

Environmental epidemiology has revealed that several organs and systems in the human body could be targets of air pollutants. Last-decade studies around the word found evidence of damage to the nervous, cardiovascular and pulmonary systems. Imaging methods have found evidence of the exposure to environmental pollutants as a risk factor in the development and complication of various diseases, as well as alterations in neurodevelopment due to prenatal exposure to air pollutants. Current imaging methods for studying the deleterious effects of pollutants include the whole spectrum of medical imaging: radiography, US, CT and MRI. Prospective studies, in addition to experimental findings, will help us to reveal the links between environmental diseases exposure and multisystem disorders. A clear understanding of the sources of heterogeneity across sites is mandatory for prediction based on imaging techniques. Medical imaging can help other fields of health sciences (advanced computation, developmental psychology, neuroscience, biomedical engineering and genetics) to improve the policies for control and monitoring to the toxic chemicals to which the population is exposed; and to early treatments when diseases appear.

## Data Availability

No applicable.

## Abbreviations

AD: Alzheimer Disease
AMRI: Anatomical magnetic resonance imaging
ARDS: Adult respiratory distress syndrome
CDE: Common data elements
CNS: Central nervous system
CO_2_: Carbon dioxide
CPF: Chlorpyrifos
CT: Computed tomography
DTI: Diffusor tensor imaging
fMRI: Functional magnetic resonance imaging
Glu: Glutamate
HMLD: Hard metal lung disease
HRCT: High-resolution computed tomography
ILO: The International Labour Organisation
MI: Myocardial infarction
MRI: Magnetic resonance imaging
MRS: Magnetic resonance spectroscopy
NAA: *N*-acetyl aspartate
NO_2_: Nitrogen dioxide
O_3_: Ozone
PAHs: Polycyclic aromatic hydrocarbons
PD: Parkinson disease
PM: Particulate matter
SO_2_: Sulfur dioxide
TRAP: Traffic-related air pollution
US: Ultrasonography
